# A Unique Dermoscopy Pattern of Solitary Cutaneous Reticulohistiocytosis

**DOI:** 10.1155/2013/674896

**Published:** 2013-02-06

**Authors:** Felipe Ladeira de Oliveira, Letycia Lopes Chagas Nogueira, Gabriel Monteiro de Castro Chaves, Mariana Daflon Vinhosa Muniz, Renata Pinto Fernandes Timbó, Mariana Martins Sasse, Carolina Degen Meotti

**Affiliations:** Laboratório Anatomical-Lab Rio de Janeiro, Sala 1115, Avenue N. S. Copacabana 680, 22050-900 Rio de Janeiro, RJ, Brazil

## Abstract

Histiocytosis represents a group of rare disorders characterized by nonneoplastic proliferation of histiocytes and solitary cutaneous reticulohistiocytosis (SCR) is a form of non-Langerhans histiocytosis. SCR consists of small papule, usually single, varying in color from yellow to brownish-red, more frequent in young adults, and uncommon in childhood. We report a case of SCR in an 11-year-old child and emphasize a unique dermoscopy pattern of this lesion and its correlation with clinical and histopathological aspects in the diagnosis.

## 1. Introduction

Initially described by Zak in 1959, solitary cutaneous reticulohistiocytosis (SCR) is a form of reticulohistiocytosis that is a rare presentation of non-Langerhans cell histiocytosis [1]. Despite being known since the 1950s its origin is still unknown. However, it is considered a nonneoplastic lesion, reactive, that originates by proliferation and differentiation of an anomalous histiocytic clone [2]. Still, regarding the etiology of the lesion, its development has already being related to traumatic areas, but the majority of cases appear spontaneously [3]. 

 Recently, some authors have reviewed a high number of similar cases and proposed the name solitary epithelioid histiocytoma instead of the archaic “reticulohistiocytoma” [4].

 It is more common in young adults and slightly more predominant in men [3]. The aim of this paper is to emphasize a unique dermoscopy pattern of this lesion and its correlation with clinical and histopathological aspects in the diagnosis.

## 2. Case Presentation

**Figure 1:**
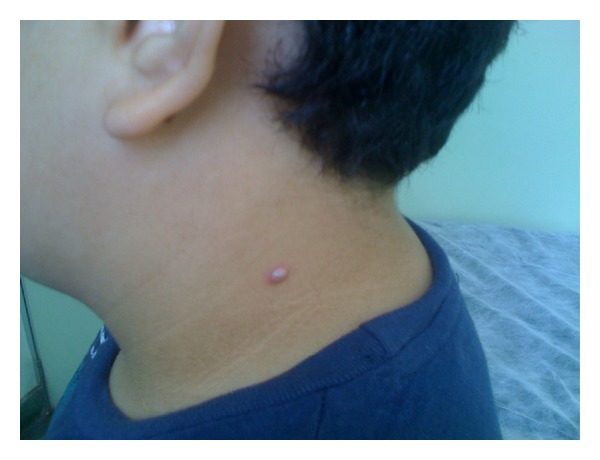
Well defined papule of firm consistency on neck.

**Figure 2:**
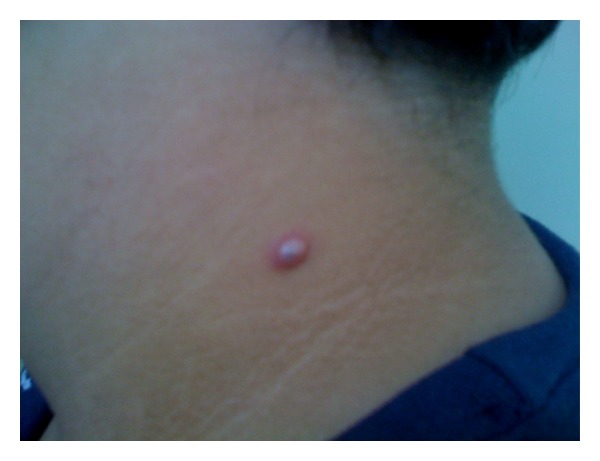
Detail of the lesion: observe the nonexudative papule.

An 11-year-old male was taken to a primary care because his mother noticed a lesion painful to touch on his neck that appeared 30 days previously; the lesion had been gradually growing. There was no history of obesity or dyslipidemia. The examination revealed a well-defined papule of firm consistency, 6.5 mm in diameter, erythematous, and nonexudative (Figures 1 and 2). There were no palpable lymph nodes or other systemic symptoms.

**Figure 3:**
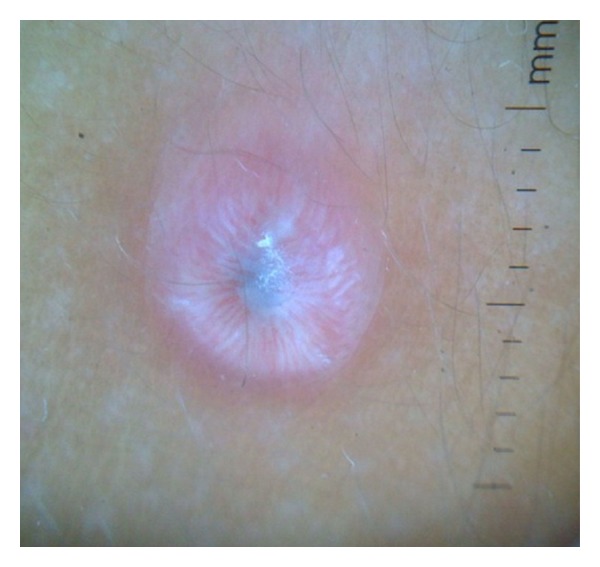
Central white-grayish area and white-pinkish streaks situated at the periphery, without dilated vessels.

**Figure 4:**
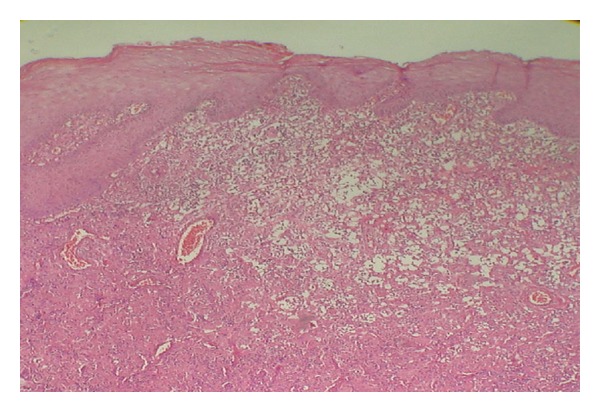
Crust on normal epidermis. Papillary and reticular dermis with edema and dilated venules and capillaries. Defined mass of eosinophilic cells in the reticular dermis.

**Figure 5:**
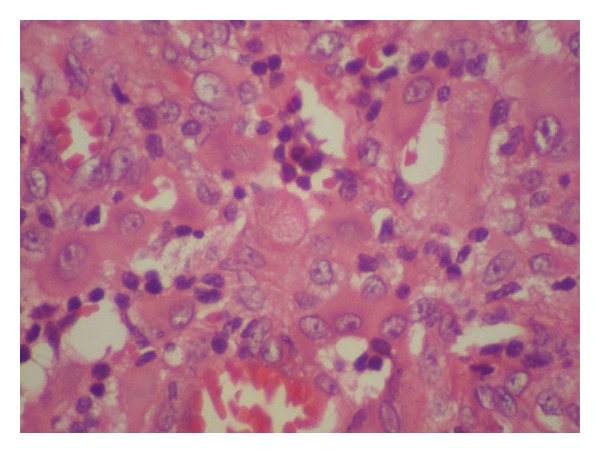
Dilated venules and capillaries with erythrocytes inside. Histiocytes with an eosinophilic glassy cytoplasm and vesiculosus nuclei. Also, lymphocytes and eosinophils were seen intermingled.

 Dermoscopy under nonpolarized light showed a well-defined central white-grayish area and white-pinkish streaks situated at the periphery, without dilated vessels (Figure 3). An excisional biopsy was done and the histopathologic study demonstrated a well-delimited lesion in the dermis that presented dilated venules and capillaries with erythrocytes inside: histiocytes with an eosinophilic cytoplasm of “glassy” appearance and vesiculosus nuclei. Also, lymphocytes were seen intermingled (Figures 4 and 5). Immunohistochemically, the neoplastic cells were positive for factor XIIIa (dentrocyte marker) and vimentin and negative for S-100 and CD34. As such, the diagnosis of SCR was established.

## 3. Discussion

SCR is characterized by an exophytic neoformation with rapid growth that has become a well-delimited papule or nodule, with a smooth surface of 0.3 to 2 centimeter in diameter. The lesions are of firm consistency and variable color, ranging from yellow to red-brownish [3]. Regarding location, it occurs more frequently on the face, neck, and superior portion of the trunk, more common in young adults [5]. Pediatric cases, as the patient reported, are rare [6]. 

 Reticulohistiocytosis can also present as diffuse cutaneous reticulohistiocytosis and multicentric reticulohistiocytosis (MR) [3]. The last refers to a rare systemic disease featuring multiple cutaneous histiocytic lesions located mainly in acral sites and on the face, involving synovial membranes and accompanied by erosive arthritis [4]. This condition has also been described in association with malignancy, suggesting that some cases can have a paraneoplastic nature [7]. Our patient had a single lesion, without articular manifestations, so the other forms were excluded. 

 The histopathological study was done objecting to exclude possible differential diagnosis. SCR differs from conventional granulomatous inflammation by being free of well-formed spherical granulomas and the presence of large epithelioid histiocytes with eosinophilic “glassy” cytoplasm [4]. The last feature is unique and common in SCR [2].

This lesion can be difficult to distinguish clinical and histopathological from solitary juvenile xanthogranuloma: however, the last classically contains lipids in the histiocytes and Touton-type histiocytic giant cells [4], both not found in the related patient's exam. 

 In its turn, dermoscopy of SCR, previously described only twice in the literature, can have an auxiliary role in its diagnosis. The dermoscopic patterns reported were different from the one observed in our patient. One study showed dotted vessel and light-brown globules on a yellow background and the other a uniformly yellow central area and a pink-orange color in the periphery [8]. The second case showed aspects similar to the juvenile xanthogranuloma, which has been called the sign of the “setting sun” and is considered indicative of the presence of xanthomatous histiocytes [8, 9]. One study demonstrated three different patterns of the reticulohistiocytomas in the same patient with multicentric reticulohistiocytosis (brown reticular structures, central white scar-like patches, and the “setting-sun” pattern) [10]. In the presented case, we suggest that the dilated venules and capillaries with erythrocytes inside observed on histology correlate with the white-pinkish streaks situated at the periphery, visibly demonstrating a degree of vessel congestion. 

 Other differential diagnoses include nodular tenosynovitis, fibrolipoma, Spitz's nevus, mastocytoma, keratoacanthoma, Hashimoto, and Pritzker's disease and pyogenic granuloma [2, 3]. These differential diagnoses were ruled out by the typical eosinophilic “glassy” cytoplasm and immunohistochemical study.

 Therefore, this case reinforces this new dermoscopy finding and the use of dermoscopy in nonpigmented skin disorders evaluation and its importance as an adjuvant in diagnosing non-Langerhans cell histiocytosis, contributing to exclude potential differential diagnosis when used together with histopathology.
